# Repeated Pregnancies With Full Trisomy 21: A Case Report and Literature Review

**DOI:** 10.7759/cureus.83275

**Published:** 2025-04-30

**Authors:** Yuri Hasegawa, Shoko Miura, Ai Nagata, Koh-Ichiro Yoshiura, Kiyonori Miura

**Affiliations:** 1 Department of Obstetrics and Gynecology, Nagasaki University Graduate School of Biomedical Sciences, Nagasaki, JPN; 2 Department of Human Genetics, Nagasaki University Graduate School of Biomedical Sciences, Nagasaki, JPN; 3 Leading Medical Research Core Unit, Nagasaki University Graduate School of Biomedical Sciences, Nagasaki, JPN

**Keywords:** genetic counseling, gonadal mosaicim, recurrent trisomy 21, trisomy 21, whole exome analysis

## Abstract

We report a rare case of a woman who had at least five pregnancies with full trisomy 21, despite being younger than 35 years. Chromosome testing was performed once on a newborn, three times on amniotic fluid, and three times on miscarriage samples. There were five cases of trisomy 21, one case of trisomy 16, and one case of a normal karyotype. Chromosome tests were performed on the patient and one of her partners to search for the cause, but they were found to be normal karyotypes. In addition, the patient and her parents’ blood samples were collected and analyzed for whole-exome analysis (trio analysis), but the cause of the recurrent trisomy was unknown. The most likely cause of recurrent trisomy 21 was gonadal mosaicism in the patient.

## Introduction

Down syndrome (trisomy 21) is the most common chromosomal abnormality. Trisomy 21 occurs in 0.45% of clinically observed pregnancies [1]. However, with the widespread use of prenatal diagnosis, recent reports have shown that trisomy 21 is found in one in 691 live births (0.14%) in the United States [2] and in 22 out of 10,000 live births (0.22%) in Japan. Prenatal genetic testing is gradually becoming more widespread in Japan, with 20% of trisomy 21 cases diagnosed prenatally in 2016 [3]. In 2016, approximately 70% of children with trisomy 21 were born to women of advanced maternal age [3]. The incidence of trisomy 21 usually increases with increasing maternal age [4]. In addition, the risk of trisomy 21 recurring in families with affected children is 1%-2% [4]. There are only a limited number of case reports of trisomy 21 being repeated three or more times, and it is extremely rare [5,6]. Possible causes for young women repeatedly becoming pregnant with children with trisomy 21 include the possibility of the mother having a slight mosaic trisomy 21 or gonadal mosaicism [5,6]. We report a rare case of a mother under 35 years who had at least five pregnancies with full trisomy 21.

## Case presentation

**Figure 1:**
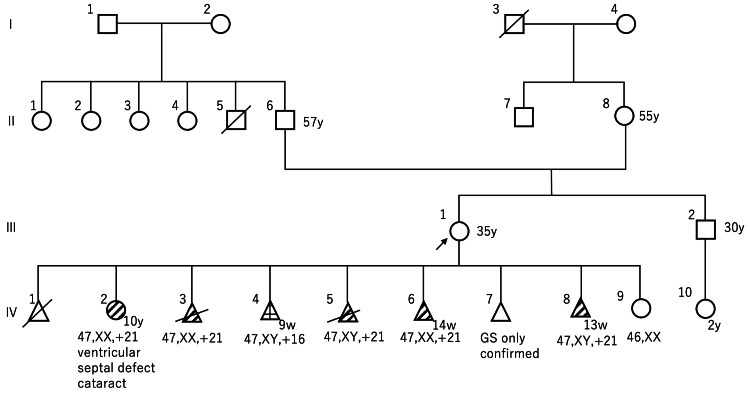
Family tree III-1 is the patient. There was no consanguineous marriage. IV-2, -3, -5, -6, and -8 had trisomy 21. IV-4 had trisomy 16. The patient had four different partners; IV-1 was with the first partner, IV-2, -3, and -4 were with the second, IV-5 and -6 were with the third, and IV-7, -8, and -9 were with the fourth. III indicates the client's own generation, and IV indicates the client's children's generation. GS: gestational sac Circle: female; square: male; black arrow: patient; diagonal line across a triangle: artificial abortion; triangle: miscarriage; diagonal line in a circle or triangle: trisomy 21; grid pattern inside a triangle: trisomy 16.

The patient was 31 years old when she made her first visit to our hospital early in her pregnancy (IV-6 of the family tree is shown in Figure 1). There were no consanguineous marriages. In nine pregnancies, she had four different partners; IV-1 was with the first partner, IV-2, -3, and -4 were with the second, IV-5 and -6 were with the third, and IV-7, -8, and -9 were with the fourth. Table 1 summarizes the number of pregnancies, partners, and chromosome tests.

**Table 1:** List of the number of pregnancies, partners, and chromosome tests AA: artificial abortion

Number of pregnancies	Age of pregnancy	Partners	Chromosome tests	Results of chromosome tests	Outcome
1	20	A	None	-	Miscarriage
2	24	B	Child’s blood	47,XX,+21	Currently alive
3	25	B	Amniotic fluid	47,XX,+21	AA
4	25	B	Chorionic villi	47,XX,+16	Miscarriage
5	31	C	Amniotic fluid	47,XX,+21	AA
6	32	C	Chorionic villi	47,XX,+21	Miscarriage
7	33	C	None	-	Miscarriage
8	34	D	Chorionic villi	47,XX,+21	Miscarriage
9	35	D	Amniotic fluid	46,XX	Currently alive

At her first visit to our hospital, she had already had three previous pregnancies with children with trisomy 21. Therefore, she requested genetic counseling and an amniotic fluid chromosome test. However, she miscarried at 14 weeks’ gestation and was also diagnosed with trisomy 21 on a chromosome examination of trophoblastic tissue. In subsequent pregnancies, she had repeated pregnancies with trisomy 21. In five of the six pregnancies for which chromosome testing was finally available, she carried a child with trisomy 21. Her first child with trisomy 21, IV-2, had a postnatal chromosome test result of 47,XX,+21 and was diagnosed with Down syndrome. This child has a ventricular septal defect and cataracts in both eyes. The maternal ages at the time of the first to ninth pregnancies were 20, 24, 25, 25, 31, 32, 33, 34, and 35 years, respectively. The first, fourth, sixth, seventh, and eighth pregnancies were miscarriages. Chromosome tests were not performed at the time of the first (IV-1) artificial abortion and seventh (IV-7) miscarriage. Chromosome tests of the chorion were performed at the time of the fourth (IV-4), sixth (IV-6), and eighth (IV-8) miscarriages, and showed 47,XY,+16 in the fourth, 47,XX,+21 in the sixth, and 47,XY,+21 in the eighth. The third (IV-3) and fifth (IV-5) pregnancies did not result in miscarriage. Amniotic fluid chromosome tests were performed at 16 weeks’ gestation in these pregnancies and showed 47,XX,+21 and 47,XY +21, respectively. After aborting the fifth pregnancy, the patient submitted chromosome tests for herself and her partner (30 cells were analyzed), both of which showed a normal karyotype.

To search for the cause of repeated trisomy 21, whole-exome sequencing (WES) was performed after abortion of her fifth pregnancy. Genomic DNA was extracted from peripheral blood samples obtained from the patient (III-1) and her parents (II-6 and II-8) (trio analysis). To perform WES (SureSelect XT AUTO HUMAN ALL Exon V5 (Agilent, Santa Clara, CA, USA); HiSeq 2500 platform (Illumina, San Diego, CA, USA); Rapid PE Cluster Kit-HS; HiSeq Rapid SBS Kit-HS (200 cycles)), paired-end analysis was performed. Trio-based WES of the patient and her parents showed no pathogenic or likely pathogenic variants with homozygous, heterozygous, and X-linked inheritance. WES was performed as follows. Sequencing reads were mapped to the human reference genome (hg19) using Novoalign (version 3.03.02; Anaconda, Inc., Austin, TX). Variant calling was conducted using the Genome Analysis Toolkit (GATK, version 3.5; Brenda Genetics, Brescia, Italy) according to the GATK Best Practices. Variant annotation was performed with ANNOVAR (downloaded on December 14, 2014). Variants meeting all of the following criteria were extracted: rare variants, defined as those with a minor allele frequency (MAF) of ≤0.5% in both the ToMMo 1KJPN and ExAC03 databases. Nonsynonymous or truncating variants located in genes defined by GENCODE Basic V19. The mean depth of coverage after PCR duplicate removal within the SureSelect V5 target region was as follows: 1) Proband (NGAK_IRUD_15_0003): 54.31×, 2) father: 130.04×, and 3) mother: 97.99×. In conclusion, no genetic variants were found that could explain any of the pregnancies that resulted in children with trisomy 21.

After the patient’s ninth pregnancy, an amniotic fluid chromosome test was performed at 17 weeks and 0 days of gestation, and it showed 46,XX. She had a spontaneous vaginal delivery at 38 weeks and 2 days of gestation. The neonate was a 2,896-g girl, and she is still in good health.

Genetic counseling

Genetic counseling was conducted several times from the first visit because of the repeated trisomy 21. We explained to the patient that neither she nor her partner had any chromosomal abnormalities, that she had repeated pregnancies resulting in a child with trisomy 21 even though her partner had changed, and that we could not find any causative genetic polymorphisms in WES. Therefore, gonadal mosaicism was a possibility. However, we told her that it was not possible to collect ovarian tissue just to examine gonadal mosaicism. This issue was also discussed at a genetic counseling department meeting. The conclusion was that performing surgery to biopsy ovarian tissue for the sole purpose of investigating the cause is not covered by insurance in Japan and is ethically problematic. The patient explained that she had had a miscarriage with 16 trisomy (IV-4) once, but that this was probably just a coincidence. During genetic counseling following the sixth miscarriage, she expressed feelings of sadness and anger. Regular follow-up sessions were necessary to provide support to the client.

The patient was loving and nurturing toward her first child. Although the patient would have preferred to continue pregnancy without prenatal testing, she was having prenatal tests performed owing to consideration of the feelings of her partner and her mother. Additionally, preimplantation genetic testing for aneuploidy (PGT-A) was not clinically available at the time of genetic counseling; therefore, no information was provided. Currently, because the patient has had multiple miscarriages, she is eligible for PGT-A in Japan. Therefore, she will need to be provided with information about PGT-A when she wishes to become pregnant in the future.

## Discussion

Review of repeated pregnancies of children with full trisomy 21

A PubMed search yielded just two cases of repeated complete trisomy 21 three or more times [5,6]. The results of the literature review are shown in Table 2. James et al. reported four families with full trisomy 21 [5]. Two mothers younger than 35 years were found to have trisomy 21 mosaicism of 0.6% and 3.7%, respectively, in blood chromosome tests. However, the oldest mother had no trisomy 21 mosaicism, even though more than 100 cells were examined. Therefore, the trisomy 21 was thought to be due to simple, accidental non-separation because of old age. Nielsen et al. reported a family in which the mother had six recurrent full trisomy 21 pregnancies [6]. The parents showed no blood relationship, and there were no abnormalities in the phenotype. The mother had 11 pregnancies, six of which were full trisomy 21 (either at birth or diagnosed prenatally). None of the pregnancies resulted in a healthy child. The authors collected blood, skin, and ovarian tissue from the parents and performed a chromosomal analysis. Since a 21-trisomy mosaic was found in the cells of the ovarian tissue, it was reported that gonadal mosaicism was the cause of 21 trisomy. In addition, because almost all of the children had full trisomy 21, they pointed out that there may be a cell lineage with not only trisomy 21 but also tetrasomy 21, or that the mother may be genetically predisposed to first meiotic errors.

**Table 2:** Review of the literature of cases of full trisomy 21 that occurred three or more times

Publication	Mother's age when she was pregnant with trisomy 21	Number of trisomy 21 pregnancies	Possible causes
James et al. [5]	23, 24, 29	3	Low-frequency 21 trisomy cell mosaicism in the mother (3.2%)
	28, 32, 33	3	Low-frequency 21 trisomy cell mosaicism in the mother (0.6%)
	37, 40, 43	3	Accidental non-separation due to the mother's advanced age
	36, 40, 40	3	Accidental non-separation due to the mother's advanced age
Nielsen et al. [6]	25, 26, 27, 28, 29, 31	6	Ovarian tissue of mother (trisomy 21 was found in 18% of the cells)
Our case	24, 25, 30, 31, 35	5	Possibility of gonadal mosaicism

We report a rare case of a mother who has had five pregnancies with full trisomy 21, despite being young. Generally, approximately 90% of trisomy 21 cases are due to inseparability during meiosis in the process of oogenesis, and its frequency increases with maternal age [4]. Warburton et al. [7] described that causes of repeated trisomy are related to (1) chance only due to the mother’s age-related risk, (2) gonadal mosaicism of a parent with trisomy, or (3) an increased risk of meiotic error, assuming that the parents were the same couple each time. If a mother experiences her first trisomy 21 pregnancy before she is 30 years old and continues to have subsequent pregnancies when she is under 30 years old, the risk of trisomy 21 recurring is much higher (8.2 times higher than is considered normal for that age) [7]. Therefore, experiencing trisomy 21 at a young age is more likely to involve an abnormality of maternal or oocyte origin (gonadal mosaicism or a predisposition to meiotic errors) than chance [7].

In the present case, even though the mother’s partner changed several times, she repeatedly had pregnancies with trisomy 21. Therefore, the cause of the trisomy 21 was clearly related to the mother. To identify the cause, the patient and her third partner underwent chromosomal testing, and WES was also performed on the patient and her parents. The patient’s chromosome test was normal. Therefore, the mother was unlikely to be a trisomy 21 mosaic. However, the chromosome test only tested 30 cells from peripheral blood lymphocytes, and the possibility remains that a small amount of trisomy 21 mosaicism is present [8]. In order to detect low-frequency trisomy 21 mosaicism in maternal blood, it is necessary to examine more than 100 cells. In this regard, it must be said that detection was insufficient. In addition, no mutations that cause disease were found in any of the homozygotes, heterozygotes, or X-linked cases in WES. Studies using WES and WGS data have shown that the sensitivity of mosaic event detection depends on the size, type, clonality, and sequencing depth of the event [9]. This also supports the low sensitivity of WES in detecting low-frequency mosaic mutations. WES may detect genetic factors that increase the likelihood of non-disjunction during maternal meiosis. For example, mutations in genes involved in chromosome segregation (e.g., REC8, SYCP3, SMC1B, MLH1, MSH5) have been reported to be associated with this condition. All cases of trisomy 21 were complete, and none were mosaics. The mother is unlikely to have a mosaic form of trisomy 21. Therefore, the most likely cause of trisomy 21 in this case was gonadal mosaicism. In cases similar to this one, performing PGT-A can help avoid unnecessary miscarriage surgeries or induced abortions. This information is crucial in genetic counseling. However, it is important to explain that reproductive assistance such as ovulation induction is necessary for women capable of natural conception.

## Conclusions

We report a rare case of full trisomy 21 that occurred five times (four of the pregnancies occurred before the mother was 35 years of age). The cause of trisomy 21 was unknown even with WES, but based on the mother’s age and other factors, the cause of the repeated trisomy 21 was likely to be gonadal mosaicism. If the client wishes to become pregnant in the future, it is desirable to provide information about PGT-A.
